# The Q175 Mouse Model of Huntington’s Disease Shows Gene Dosage- and Age-Related Decline in Circadian Rhythms of Activity and Sleep

**DOI:** 10.1371/journal.pone.0069993

**Published:** 2013-07-30

**Authors:** Dawn H. Loh, Takashi Kudo, Danny Truong, Yingfei Wu, Christopher S. Colwell

**Affiliations:** Laboratory of Circadian and Sleep Medicine, Department of Psychiatry and Biobehavioral Sciences, University of California Los Angeles, Los Angeles, California, United States of America; Kent State University, United States of America

## Abstract

Sleep and circadian disruptions are commonly reported by patients with neurodegenerative diseases, suggesting these may be an endophenotype of the disorders. Several mouse models of Huntington’s disease (HD) that recapitulate the disease progression and motor dysfunction of HD also exhibit sleep and circadian rhythm disruption. Of these, the strongest effects are observed in the transgenic models with multiple copies of mutant huntingtin gene. For developing treatments of the human disease, knock-in (KI) models offer advantages of genetic precision of the insertion and control of mutation copy number. Therefore, we assayed locomotor activity and immobility-defined sleep in a new model of HD with an expansion of the KI repeats (Q175). We found evidence for gene dose- and age-dependent circadian disruption in the behavior of the Q175 line. We did not see evidence for loss of cells or disruption of the molecular oscillator in the master pacemaker, the suprachiasmatic nucleus (SCN). The combination of the precise genetic targeting in the Q175 model and the observed sleep and circadian disruptions make it tractable to study the interaction of the underlying pathology of HD and the mechanisms by which the disruptions occur.

## Introduction

Huntington’s disease (HD) is characterized by motor dysfunction and cognitive decline, and is caused by an autosomal dominant expansion of CAG repeats in the *Huntingtin* (*HTT*) gene [1]. HD patients present primarily with involuntary movements (chorea) and loss of motor control. It is invariably fatal, age of onset varies with the severity of the mutation, and there are no significant treatment options to date [2], [3]. In addition to the motor dysfunction, HD patients often present with non-motor symptoms that include cognitive dysfunction [4], [5], affective disorders [6]–[8] and sleep and circadian rhythm disruptions [9]–[12], which can all precede the onset of chorea. As sleep and circadian disruptions can themselves lead to similar non-motor symptoms like mood disorders and cognitive dysfunction (e.g. [13]–[16]), it is vital to parse out the relative contribution of sleep and circadian rhythm disruptions.

To study the underlying mechanisms of sleep-wake disturbances associated with HD, we first need to identify suitable animal models that recapitulate as many symptom sets of HD as possible. While there are numerous mouse models of HD, no single model has yet been determined to be the ideal mirror of human HD [17]. Two examples of transgenic insertion HD mouse models are the exon 1-fragment model (R6/2 [18]) and the stable 90Q-repeat with full-length human *HTT* gene model (BACHD [19]). Circadian deficits begin in the R6/2 line at 10 weeks, starting with imprecise activity onset and progressing to a complete loss of rhythms in activity [10], [20]. The BACHD model exhibits a decline in the power of circadian rhythms of activity at 3 months [20], [21]. Of the current complement of HD mouse models, the knock-in of 140 CAG repeats into exon 1 of the mouse *Htt* gene (CAG140 KI [22], [23]) has considerable benefits as the genetically precise insertion of the mutation into the *Htt* locus rules out position and copy number effects that may affect the other transgenic models. The age-related decline in circadian rhythms in the CAG 140 KI line was not distinguishable from the age-related decline also observed in WT mice at 12 months of age, suggesting that the effects of the targeted CAG repeats are subtle in the heterozygote mutants [20]. A spontaneous expansion mutant that arose in this line of mice, with over 175 CAG repeats (Q175), has motor and cognitive deficits that have earlier onsets than the CAG140 line [24], and thus may also exhibit sleep and circadian rhythm disruption that can be detected before WT rhythms also decline.

We examined the spontaneous expansion Q175 model for sleep and circadian rhythm disruptions over the course of 12 months of age. We also examined the decline in motor function and the impact of the mutation on circadian gene expression in the SCN.

## Methods

### Animals

The experimental protocols used in this study were approved by the UCLA Animal Research Committee (ARC 2009-022), and all recommendations for animal use and welfare, as dictated by the UCLA Division of Laboratory Animal Medicine and the guidelines from the National Institutes of Health, were followed. A line of mice with a spontaneous expansion of the CAG repeats in the CAG140 KI line, the Q175 mutation, was obtained from the CHDI colony of Q175 mice at the Jackson Laboratories (Bar Harbor, Maine). The Q175 mice have previously been shown to have around 188 CAG repeats [24]. We obtained an age-matched cohort of male mice on the C57Bl6/J background that were wild-type (WT), heterozygote (Het) and homozygote (Hom) for the Q175 allele.

### Monitoring of Locomotor Activity

Adult male mice (WT *n* = 8, Q175 Het *n* = 8, Q175 Hom *n* = 8) at 8 weeks of age were singly housed in cages containing wheels (11.5 cm diameter, Mini Mitter, Bend, OR), and locomotor activity was recorded as previously described [20], [25], [26]. Mice were entrained to a 12∶12 hr light:dark (LD) cycle for a minimum of 2 weeks prior to collection of 10–14 days of data under LD conditions, followed by 10–14 days in constant darkness (DD) to obtain free-running activity. The behavioral response to a phase delaying 10 min pulse of light (100 lux at cage level) at circadian time (CT) 16 was measured as previously described [25], [26]. Following these assays, the mice were entrained to 12∶12 LD for a minimum of 14 days. Mice were then exposed to 10–14 days of a skeleton photoperiod of 1∶11:1∶11 LDLD to determine relative entrainment to morning versus evening photic cues. The mice were continuously housed in the same environment and subjected to the same battery of lighting conditions and assays at each age mark (3, 6, 9, and 12 months), and only removed from these conditions to record motor function (at 6, 9 and 12 months as described below) and behavioral sleep (at 9 and 12 months).

Wheel revolutions were recorded in 3 min bins, and 10 days of data under each condition were averaged for analysis. Free-running period (tau, τ) was determined using the χ^2^ periodogram [27] and the power of the rhythm was determined by multiplying the amplitude, *Qp*, by 100/*n*, where *n* = number of datapoints examined (e.g. [28]) using the El Temps program (A. Diez-Noguera, Barcelona, Spain). Activity amount was determined by averaging 10 days of wheel revolutions (rev/hr). Activity duration (alpha, α) was determined by the duration of activity over the threshold of the mean using an average waveform of 10 days of activity. Nocturnality was determined from the average percentage of activity conducted during the dark. Precision was determined by calculating the daily variation in onset from a best-fit regression line drawn through 10 days of activity in both LD and DD conditions using the Clocklab program (Actimetrics, Wilmette, IL). Fragmentation was defined by bouts/day, where a bout was determined as 21 consecutive minutes of activity (maxgap setting of 21 min).

### Motor Function Tests

We employed both the accelerating rotarod test [29] and challenging beam test [30] to determine the progression of motor dysfunction in the mice. All tests were performed at the light:dark transition (ZT 11 to 12; one hour prior to lights-off) to minimize effects of sleep deprivation.

At 6, 9 and 12 months, the mice were trained on the rotarod (Ugo Basile, Varese, Italy) with 5 trials on the first day, as previously described [29]. The accelerating rotarod went from 5 to a maximum of 38 rpm and the maximum length of each trial was 600 sec. On the second day, mice were placed on the rotarod and the latency to fall from the rotarod was recorded from 5 trials, and averaged between trials.

We employed a modified version of the traditional beam walking test, as described in detail by Fleming and colleagues [30]. The premise of this task, named the challenging beam test, is to determine the ability of mice to cross a bridge of decreasing width, with re-entry into the home cage as the motivating factor [30]. The beam narrows in 4 intervals from 33 mm >24 mm >18 mm >6 mm, with each bridge spanning 253 mm in length. Mice at 9 and 12 months of age were trained on the beam for 5 consecutive trials at ZT 11 on two consecutive days. During each trial, each mouse was placed on the widest end of the beam and allowed to cross with minimal handling by the experimenter. On the third day, the mice were further challenged during testing by the application of a metal grid (10×10 mm spacing) overlaid onto the bridge. A second experimenter was on hand to record the progress of the animal using a hand held camcorder and five consecutive trials were recorded. The videos were scored by two independent observers blind to the experimental conditions for the time to cross the beam and touch the home cage, the number of steps taken by one designated limb, and the number of mis-steps (errors) made by each mouse. We considered an error to have occured when more than half of the foot in question dipped below the grid. Time to cross, number of steps, and number of errors were averaged across the 5 trials per mouse to give the final reported values.

### Video Measurement of Immobility-defined Sleep

Mice were habituated to see-through plastic cages containing bedding, but without the addition of nesting material, for a minimum of 3 days prior to video recording of behavior. Mice were housed on a 12∶12 LD cycle in keeping with their previous housing conditions at 9 and 12 months of age. A side-on view of each cage was obtained, with minimal occlusion by the food bin or water bottle, both of which were top-mounted. Video capture was accomplished using surveillance cameras with visible light filters (Gadspot Inc., City of Industry, CA) connected to the video-capture card (Adlink Technology Inc., Irvine, CA) on a Dell Optiplex computer system. The AnyMaze software (Stoelting Co., Wood Dale, IL) was used to track the animals as described by Fisher and colleagues [31], who found 99% correlation between immobility-defined and EEG-defined sleep using an immobility detection threshold set to 95% of the area of the animal immobile for 40 seconds. Immobility-defined sleep in this study is thus defined as 95% immobility recorded in an animal for a minimum of 40 seconds. Continuous recording and tracking of the mice under a 12∶12 LD cycle was performed for a minimum of 3 days, with randomized visits (1/day) by the experimenter to confirm mouse health and video recording. We used data collected from days 2 and 3 for further analysis. Immobility-defined sleep data were exported in 1 min bins, and total sleep was determined by summing the duration of sleep in the day (ZT 0–12) or night (ZT 12–24). Number of sleep bouts was determined using Clocklab at the resolution of 1 min bins of Anymaze data (minimum of 40 sec sleep per bin). Average bout duration (number of consecutive bins with at least 40 sec of sleep per bin) was determined within day or night. An average waveform of hourly sleep from both days was produced per genotype per age group for the purpose of graphical display. To address concerns that sleep measurements may be over- or under-estimated in Q175 mutants, we analyzed the data at 90 and 97% immobility, which have previously been correlated by Fisher and colleagues [31] to simultaneous EEG recordings as over-estimating sleep by 6 min per hour and under-estimating sleep by 6 min per hour respectively. In our hands, these altered thresholds changed the amount of sleep measured, but did not change the overall findings (**Text S1**, **Fig. S1** and **Table S1**).

### Histology and Immunohistochemistry (IHC)

To determine cell counts and the expression of a core circadian gene, we perfused 13 month old WT, Q175 Het and Hom mice at either ZT 2 (2 hours after lights on; PER2 trough in SCN) or ZT 14 (2 hours after lights off; PER2 peak in SCN). Coronal sections (20 µm) were cut, and the sections containing the SCN were analyzed. Alternate sections of SCN were chosen for either Nissl staining to determine cell counts or IHC with an antibody against PER2 to determine the peak/trough of the molecular oscillator.

Neuronal cell bodies through the middle of the rostro-caudal axis of the SCN were identified using Nissl stain (0.1% Cresyl Violet). Sections through the SCN were photographed at 10X magnification, and counts of cell bodies within a defined area were determined by averaging the numbers from two investigators blind to the conditions. The surface area of the SCN and the lateral ventricles were determined using Axiovision (Carl Zeiss).

IHC staining for PER2 using free-floating sections was performed as previously described [20]. Coronal brain sections through the middle of the rostro-caudal axis of the SCN were chosen for IHC using affinity-purified goat polyclonal antibody raised against a peptide mapping to the N-terminal of mouse PER2 (1∶400; SC-7729; Santa Cruz Antibodies, Santa Cruz, CA). To detect PER2 staining, avidin-biotin complex detection was applied using the Vectastain ABC kit (Vector Laboratories, Burlingame, CA) and incubation with 3-3′-diaminobenzidine tetrahydrochloride and 8% nickel chloride. Images were captured with AxioVision camera systems (Carl Zeiss, Thornwood, NY). Antibody specificity was confirmed with no-primary antibody and blocking peptide control sections. To determine the number of PER2-positive (PER2+) cells, photographs were taken at 40X magnification, and cell counts were averaged between the numbers determined by two investigators blind to the conditions.

### Statistical Analysis

For comparison of WT, Q175 Het and Hom locomotor activity and sleep parameters that passed normality and equal variance tests within each age group, we applied one way analysis of variance (ANOVA) for which we report the *F* statistic, and deemed differences as significant if *P*<0.05. For any parameter that failed either normality or equal variance tests, ANOVA on ranks was applied and *H* values reported. To compare the effects of gene dosage and age, we applied two way repeated measures ANOVA. *Post hoc* Bonferroni’s *t*-test pairwise comparisons were applied in the event of significant effects of genotype or age. In the instance of failed normality or equality, Dunn’s method was used instead. Values are reported as mean ± standard error of the mean (SEM).

## Results

### Q175 Mutant Mice Show Age- and Gene Dosage-related Decline in Circadian Rhythms of Wheel Running Activity

**Figure 1:**
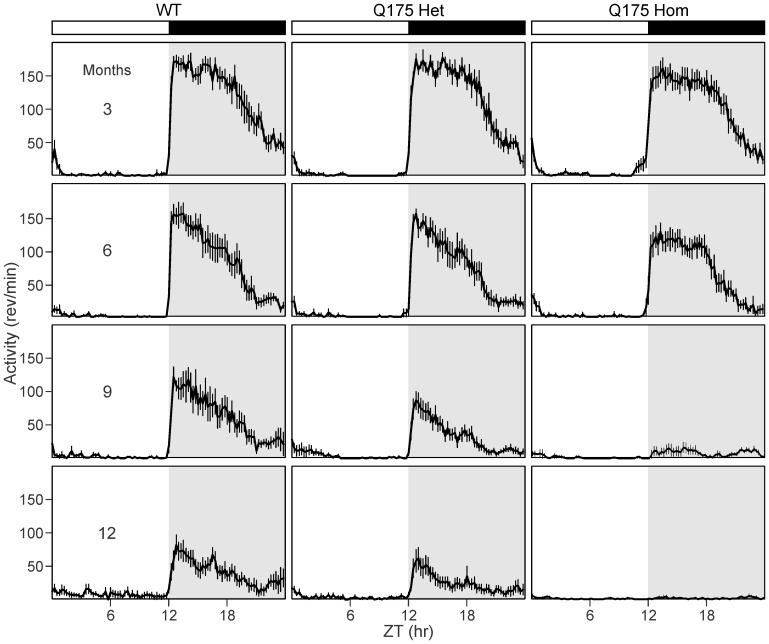
Representative waveforms of wheel running activity from 3 to 12 months of age. Average waveforms from 10 days of wheel running activity in LD are shown and standard errors across animals are indicated. The white/black bar on the top indicates the 12∶12 hr light:dark (LD) cycle, and gray shading in the waveforms indicates lights off. Sample sizes are the same as reported in **Table 1**. From left to right: WT, Q175 Het and Hom. From top to bottom: 3, 6, 9 and 12 months of age.

**Figure 2:**
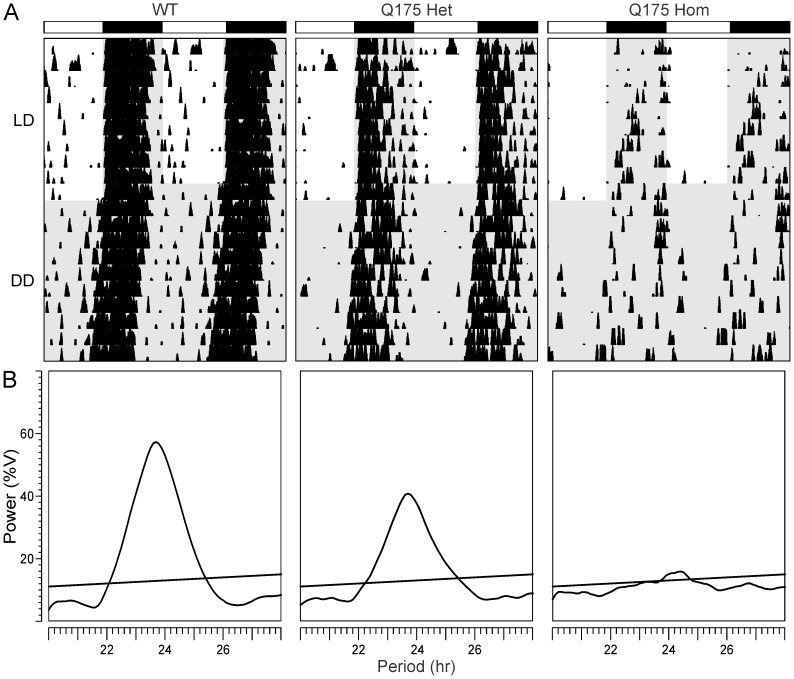
Circadian deficits in locomotor activity in Q175 mutant mice at 9 months of age. **A.** Representative double plotted actograms of wheel running activity from age-matched WT (left), Q175 Het (middle) and Q175 Hom (right) mice under 12∶12 light:dark (LD) and constant darkness (DD) are shown. The white/black bars on top indicate the LD cycle, and gray shading indicates darkness. Successive days of activity are plotted from top to bottom. **B.** Chi-square (χ^2^) periodograms of 10 days of activity in DD are shown from the three genotypes at 9 months, with the peak of the periodogram indicating the free-running period of each mouse. Power (%V) refers to the normalized amplitude of the periodogram. The intersecting diagonal line indicates significance to *P*<0.05.

**Figure 3:**
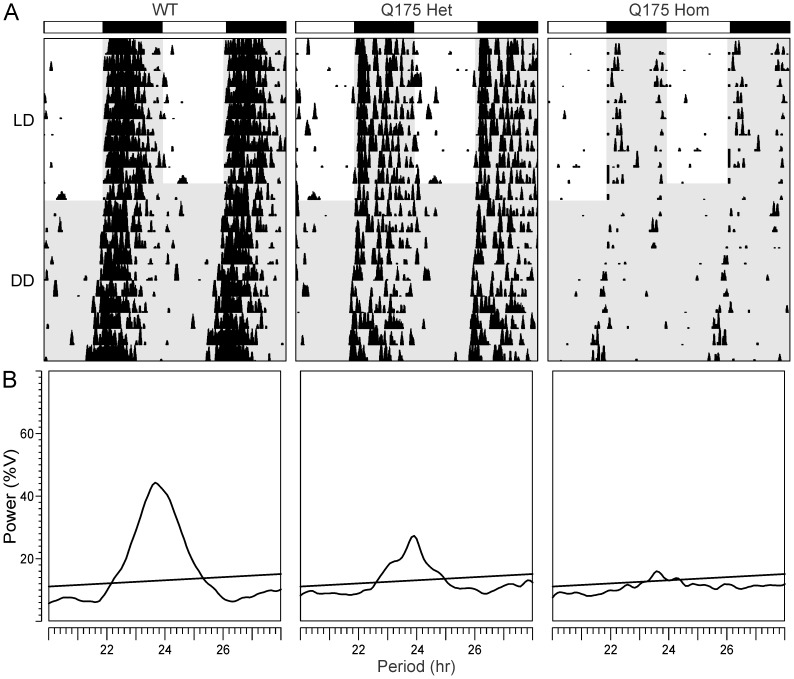
Circadian deficits in locomotor activity in Q175 mutant mice at 12 months of age. **A.** Representative double plotted actograms of wheel running activity from age-matched WT (left), Q175 Het (middle) and Q175 Hom (right) mice under 12∶12 light:dark (LD) and constant darkness (DD) are shown. The white/black bars on top indicate the LD cycle, and gray shading indicates darkness. Successive days of activity are plotted from top to bottom. **B.** Chi-square (χ^2^) periodograms of 10 days of activity in DD are shown from the three genotypes at 12 months, with the peak of the periodogram indicating the free-running period of each mouse. Power (%V) refers to the normalized amplitude of the periodogram. The intersecting diagonal line indicates significance to *P*<0.05.

**Figure 4:**
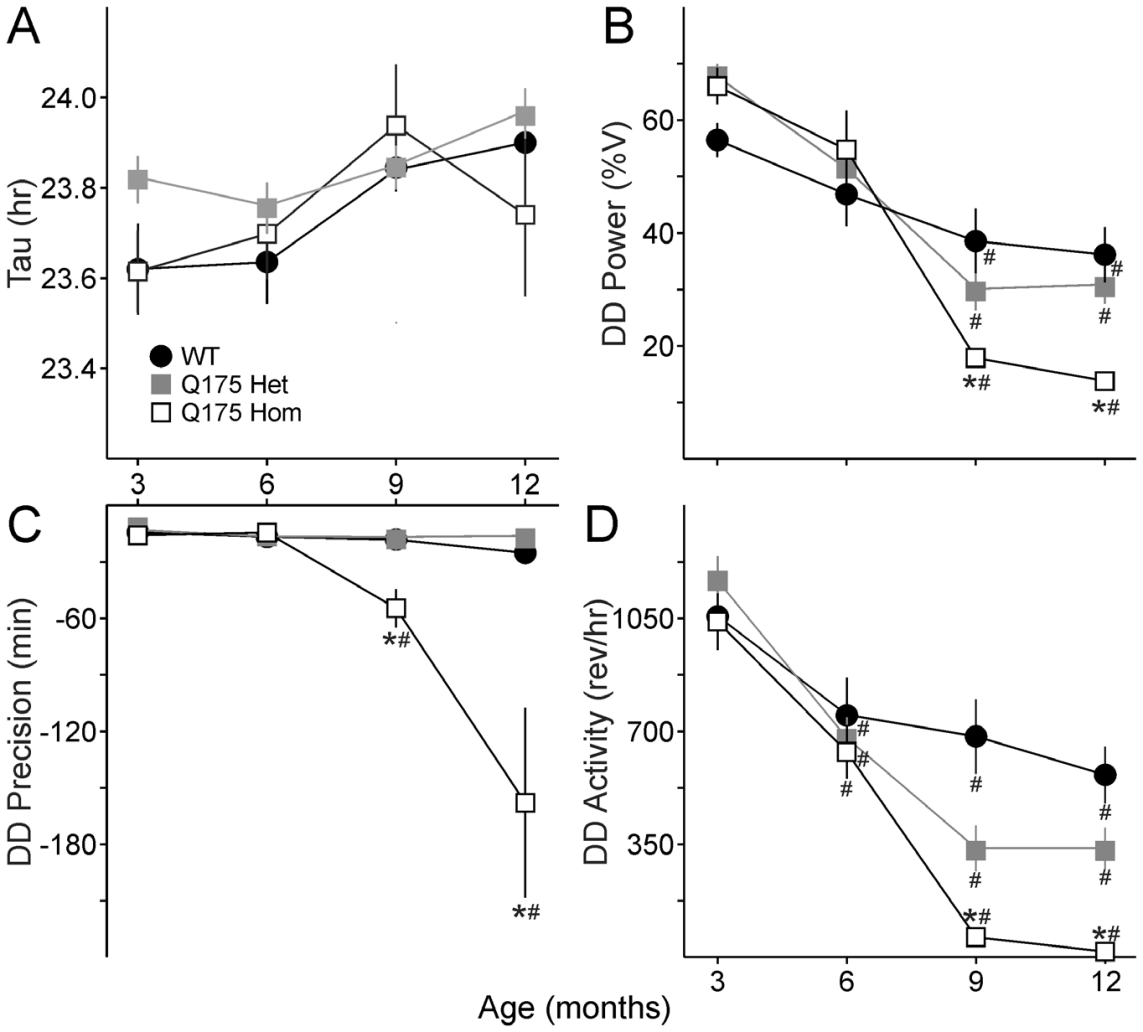
Age-related decline in circadian rhythms of locomotor activity in Q175 mutants under DD conditions. **A.** Free-running period (tau) is no different between the three genotypes. **B.** Power, as measured by the X^2^ periodogram, declines dramatically in Q175 Hom mutants at 9 months. C. The cycle-to-cycle activity onset is less precise in Q175 Hom mutants from 9 months. D. The amount of activity (wheel revolutions per hour, rev/hr) plummets in Q175 Hom mutants at 9 months. * indicates significance to *P*<0.05 in *post hoc* pairwise comparisons with WT within each age group after the two way ANOVA revealed an effect of genotype. Effect of age within each genotype is indicated with #. *F* and *P* statistics are reported in **Table 2**.

**Figure 5:**
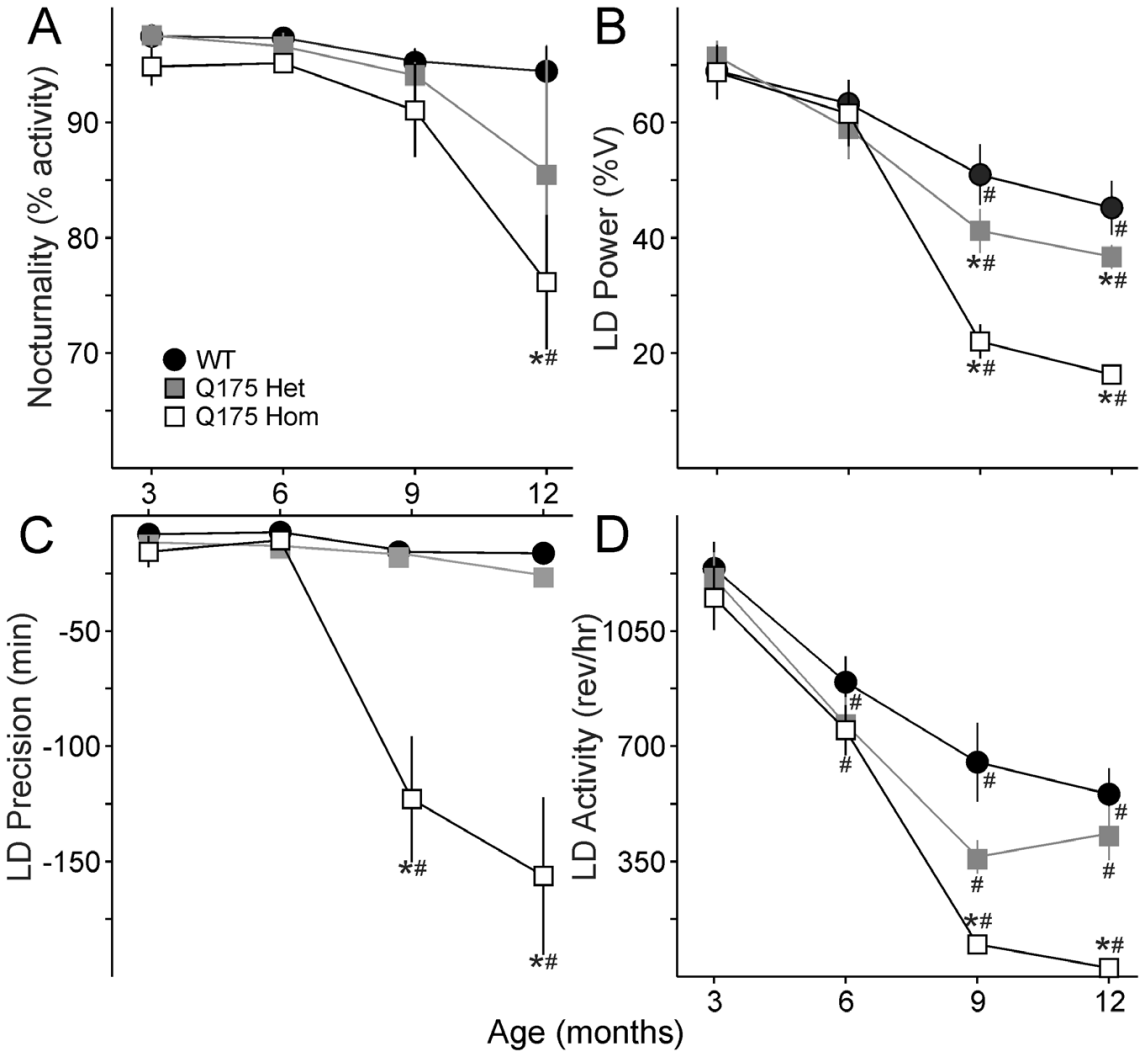
Age-related decline in rhythms of locomotor activity in Q175 mutants in LD conditions. **A.** The percentage of activity at night (nocturnality) declines at 9 months in Q175 Hom mutants. **B.** Power, as measured by the χ^2^ periodogram, declines dramatically in Q175 Hom mutants at 9 months. **C.** The cycle-to-cycle activity onset is less precise in Q175 Hom mutants from 9 months. **D.** The amount of activity (wheel revolutions per hour, rev/hr) declines sharply in Q175 Hom mutants at 9 months. * indicates significance to *P*<0.05 in *post hoc* pairwise comparisons with WT within each age group after the two way ANOVA revealed an effect of genotype. Effect of age within each genotype is indicated with #. *F* and *P* statistics are reported in **Table 2**.

We assayed an age-matched cohort of mice to determine the impact of the Q175 mutation on circadian locomotor activity from 3 to 12 months of age. Young adult (3 months and 6 months) Q175 mutant mice were not significantly different from their WT counterparts, showing equally consolidated and precise rhythms in activity that were nocturnal in nature under 12∶12 light dark (LD) conditions (**Tables 1** **,** **2**). However, as the mice aged, the strength of the rhythm (power) declined sharply in the Q175 Hom mice, and by 9 months, circadian dysfunction was apparent (**Fig. 1** **,** **2**). This was characterized by a decrease in the precision and amount of activity under both LD and constant darkness (DD) conditions (**Fig. 2A**), along with greatly reduced power of free running rhythms in DD (Fig. 2B). The age progression of circadian deficits continued at 12 months (**Fig. 3**), albeit without a change in the free-running period of the mutants (Fig. 4A). The power of the free-running rhythm in DD decreased (**Fig. 4B**) and precision of cycle-to-cycle onset worsened (**Fig. 4C**) as the Q175 Hom mice aged from 9 to 12 months. The amount of activity in the Hom mice remained low starting at 9 months and did not decline further (**Fig. 4D**). Similar genotype- and age-dependent deficits were observed under LD conditions, where the Q175 Hom mice were less nocturnal (**Fig. 5A**), had lower power rhythms (**Fig. 5B**) that were less precise (**Fig. 5C**) and an overall decline in activity amount (**Fig. 5D**).

**Table 1:** Circadian parameters of activity in LD and DD in WT, Q175 Het and Hom mice from 3 months to 12 months of age.

3 months old		WT	Q175 Het	Q175 Hom	One way ANOVA		
		*n* = 8	*n* = 8	*n* = 8	*dof*	*F*	*P*
**LD**	Nocturnality	97.5±0.7	97.5±0.9	94.8±1.5	2,23	*H* = 2.82	0.244
	Power (%V)	68.9±1.9	71.4±2.6	68.7±4.4		0.23	0.794
	Activity (rev/hr)	1239.2±75.5	1210.3±72.7	1150.3±90.7		*H* = 0.74	0.692
	Alpha, α (min)	612.9±23.4	550.9±19.0	598.3±28.5		1.83	0.185
	Fragmentation (bouts/day)	3.7±0.3	3.6±0.3	3.9±0.3		0.19	0.831
	Precision (min)	−8.1±1.6	−11.6±2.4	−15.6±6.3		*H* = 1.10	0.578
**DD**	Tau, τ (hr)	23.62±0.09	23.82±0.04	23.62±0.08		2.32	0.123
	Power (%)	56.5±2.8	67.7±2.1	65.9±3.0		5.17	0.015
	Activity (rev/hr)	1055.6±93.2	1166.0±72.4	1039.7±81.3		0.69	0.512
	Alpha, α (min)	496.6±19.8	497.4±19.6	524.4±12.5		0.81	0.460
	Fragmentation (bouts/day)	5.0±0.3	4.4±0.3	4.9±0.3		1.19	0.323
	Precision (min)	−14.3±2.4	−13.1±2.5	−15.9±2.5		0.33	0.722
							
**6 months old**		**WT**	**Q175 Het**	**Q175 Hom**			
**LD**	Nocturnality (%A in dark)	97.3±0.6	96.6±1.1	95.1±0.8	2,23	1.65	0.216
	Power (%V)	63.3±3.8	58.9±4.9	61.5±5.3		0.22	0.805
	Activity (rev/hr)	894.6±72.2	766.5±75.9	748.6±71.1		1.19	0.325
	Alpha, α (min)	544.2±25.9	465.3±29.6	529.7±23.9		2.49	0.107
	Fragmentation (bouts/day)	4.4±0.4	4.5±0.3	4.2±0.5		0.14	0.871
	Precision (min)	−7.2±1.5	−13.2±2.3	−10.6±2.2		*H* = 4.37	0.113
**DD**	Tau, τ (hr)	23.64±0.08	23.76±0.05	23.70±0.09		0.61	0.552
	Power (%)	46.9±5.3	51.4±4.4	54.7±6.5		*H* = 1.745	0.418
	Activity (rev/hr)	748.9±109.3	677.1±61.5	636.3±77.5		0.45	0.645
	Alpha, α (min)	467.3±24.4	453.9±28.7	517.9±23.7		1.73	0.201
	Fragmentation (bouts/day)	6.0±0.5	5.0±0.3	4.6±0.6		2.09	0.148
	Precision (min)	−16.8±2.5	−16.6±2.9	−14.5±1.5		0.29	0.750
							
**9 months old**		**WT**	**Q175 Het**	**Q175 Hom**			
**LD**	Nocturnality	95.3±0.9	94.1±1.1	91.0±3.8	2,22	*H* = 0.58	0.747
	Power (%V)	50.9±5.2	41.2±3.8	22.0±2.8*		*H* = 14.03	<0.001
	Activity (rev/hr)	651.5±119.7	363.7±50.0	98.2±27.1*		*H* = 15.77	<0.001
	Alpha, α (min)	529.9±25.2	423.6±47.8	462.3±32.9		2.53	0.105
	Fragmentation (bouts/day)	5.6±0.7	6.4±0.4	4.8±0.4		2.24	0.133
	Precision (min)	−15.8±2.3	−17.2±3.1	−122.9±25.5*^∧^		*H* = 14.76	<0.001
**DD**	Tau, τ (hr)	23.8±0.1	23.8±0.1	23.9±0.1		0.39	0.686
	Power (%)	38.6±5.7	30.1±3.8	17.9±1.7*^∧^		*H* = 10.28	0.006
	Activity (rev/hr)	681.7±115.5	334.6±71.3	61.9±11.7*		*H* = 14.42	<0.001
	Alpha, α (min)	609.3±47.6	523.1±32.2	496.9±62.1		1.49	0.249
	Fragmentation (bouts/day)	6.1±0.7	6.9±0.4	4.3±0.2*^∧^		8.45	0.002
	Precision (min)	−18.1±6.9	−16.8±3.5	−54.7±10.2*^∧^		9.38	0.001
							
**12 months old**		**WT**	**Q175 Het**	**Q175 Hom**			
**LD**	Nocturnality	94.4±2.2	85.7±9.7	76.1±5.4*^∧^	2,21	*H* = 9.47	0.009
	Power (%V)	45.2±4.7	36.7±2.0	16.2±1.2*^∧^		27.12	<0.001
	Activity (rev/hr)	554.3±78.2	434.6±81.0	26.4±5.9*^∧^		*H* = 14.86	<0.001
	Alpha, α (min)	635.1±22.7	595.1±82.4	516.1±65.9		*H* = 1.99	0.368
	Fragmentation (bouts/day)	5.7±0.5	6.9±0.5	4.3±0.6*		5.10	0.017
	Precision (min)	−16.3±3.5	−25.8±1.7	−156.3±31.9*		*H* = 15.56	<0.001
**DD**	Tau, τ (hr)	23.9±0.1	24.0±0.1	23.7±0.2		3.34	0.188
	Power (%)	36.2±4.9	30.8±3.4	13.8±0.6*^∧^		*H* = 15.21	<0.001
	Activity (rev/hr)	562.1±88.1	333.6±63.6	17.3±3.3*^∧^		*H* = 16.68	<0.001
	Alpha, α (min)	604.1±38.9	529.6±22.0	183.7±44.2*^∧^		*H* = 15.84	<0.001
	Fragmentation (bouts/day)	6.2±0.6	7.4±0.6	3.7±0.3*^∧^		16.34	<0.001
	Precision (min)	−25.3±2.2	−16.2±2.5	−158.0±47.2*		*H* = 16.33	<0.001

Parameters were analyzed using one-way ANOVA, and the *F* and *P* statistics are reported for genotype comparisons within each age group. *H* values are reported from ANOVA on ranks comparisons in the event of failed normality or equal variance tests. * indicates that *post hoc* Bonferroni’s *t*-tests detected significant differences compared to WT, and ^∧^ indicates significant differences were detected between Het and Hom groups.

**Table 2:** Two way repeated measures ANOVA were used to determine the effects of age and genotype in the same cohort of mice from 3 to 12 months, and we report the *F* and *P* statistics for the comparisons reported in **Fig. 4** and **5** in this table.

		Two way repeated measures ANOVA					
		Age		Genotype		Interactions	
		*F*	*P*	*F*	*P*	*F*	*P*
**LD**	Nocturnality	6.01	0.001	1.52	0.242	1.74	0.126
	Power (%V)	110.65	<0.001	8.06	0.002	8.04	<0.001
	Activity (rev/hr)	141.72	<0.001	11.26	<0.001	4.79	<0.001
	Alpha, α (min)	7.44	<0.001	3.72	0.041	0.89	0.511
	Fragmentation (bouts/day)	22.15	<0.001	3.19	0.062	3.58	0.004
	Precision (min)	5.52	0.002	6.24	0.007	2.60	0.026
							
**DD**	Tau, τ (hr)	4.61	0.006	0.83	0.451	1.13	0.355
	Power (%)	76.03	<0.001	2.17	0.139	6.51	<0.001
	Activity (rev/hr)	77.04	<0.001	13.99	<0.001	4.96	<0.001
	Alpha, α (min)	4.93	0.004	12.06	<0.001	12.19	<0.001
	Fragmentation (bouts/day)	5.86	0.001	9.21	0.001	7.95	<0.001
	Precision (min)	8.15	<0.001	11.60	<0.001	6.69	<0.001

Sample sizes are as reported in **Table 1**.

The reduced nocturnality of the Q175 Hom mice suggests deficits in the light response, which we tested by exposing the mice to discrete pulses of light to induce phase shifts in their free-running rhythms. The light-induced phase delay increased in magnitude in Q175 Hom mice, beginning at 9 months and increasing further at 12 months compared to WT and Het mice (**Table 3**). Housing mice under a skeleton photoperiod also revealed deficits in the temporal patterning of activity that began at 6 months in the Q175 Hom mice (Table 4).

**Table 3:** Increased phase delay in response to a brief pulse of light at CT 16 in Q175 mutants.

	WT	Q175 Het	Q175 Hom
	*n* = 8	*n* = 7	*n* = 8
**Phase delay (min)**			
3 months	113±5	118±6	119±4
6 months	120±5	111±4	103±6
9 months	110±2	103±2	120±12
12 months	119±5	104±2	213±24*^∧^

Data were analyzed using one way ANOVA within an age group. * indicates that *post hoc* Bonferroni’s *t*-tests detected significant differences compared to WT, and ^∧^ indicates significant differences were detected between Het and Hom groups.

**Table 4:** Reduced nocturnality of Q175 Hom mice revealed by skeleton photoperiod.

	WT	Q175 Het	Q175 Hom
	*n* = 8	*n* = 7	*n* = 8
**Activity in subjective night (%)**			
3 months	93.2±1.5	96.6±1.1	93.7±1.9
6 months	96.2±2.7	95.7±1.0	87.7±1.7*
9 months	75.8±3.3	79.5±7.4	79.8±4.1
12 months	89.3±2.0	95.4±1.7	69.6±7.1*

Data were analyzed using one way ANOVA within an age group. * indicates that *post hoc* Bonferroni’s *t*-tests detected significant differences compared to WT.

We conclude from these wheel running assays that the Q175 Hom mutants show a rapid decline of rhythms in locomotor activity from 9 months of age, with poor precision and abnormal temporal patterning of activity. The Q175 Het mutants also exhibit an age-related decline in the power of activity rhythms, but to a lesser degree than the Hom mutants, suggesting an effect of gene dosage.

### Motor Function Declines with Age in Q175 Mutant Mice

We wished to determine if the Q175 line recapitulated the HD-like symptoms observed in its preceding line, the CAG140 knock-in model. As such, we performed two motor function tests previously found robust enough to detect motor deficits in mouse models of neurodegeneration: the accelerating rotarod test (e.g. [28]) and a modified version of the beam walking test: the challenging beam test [29].

**Figure 6:**
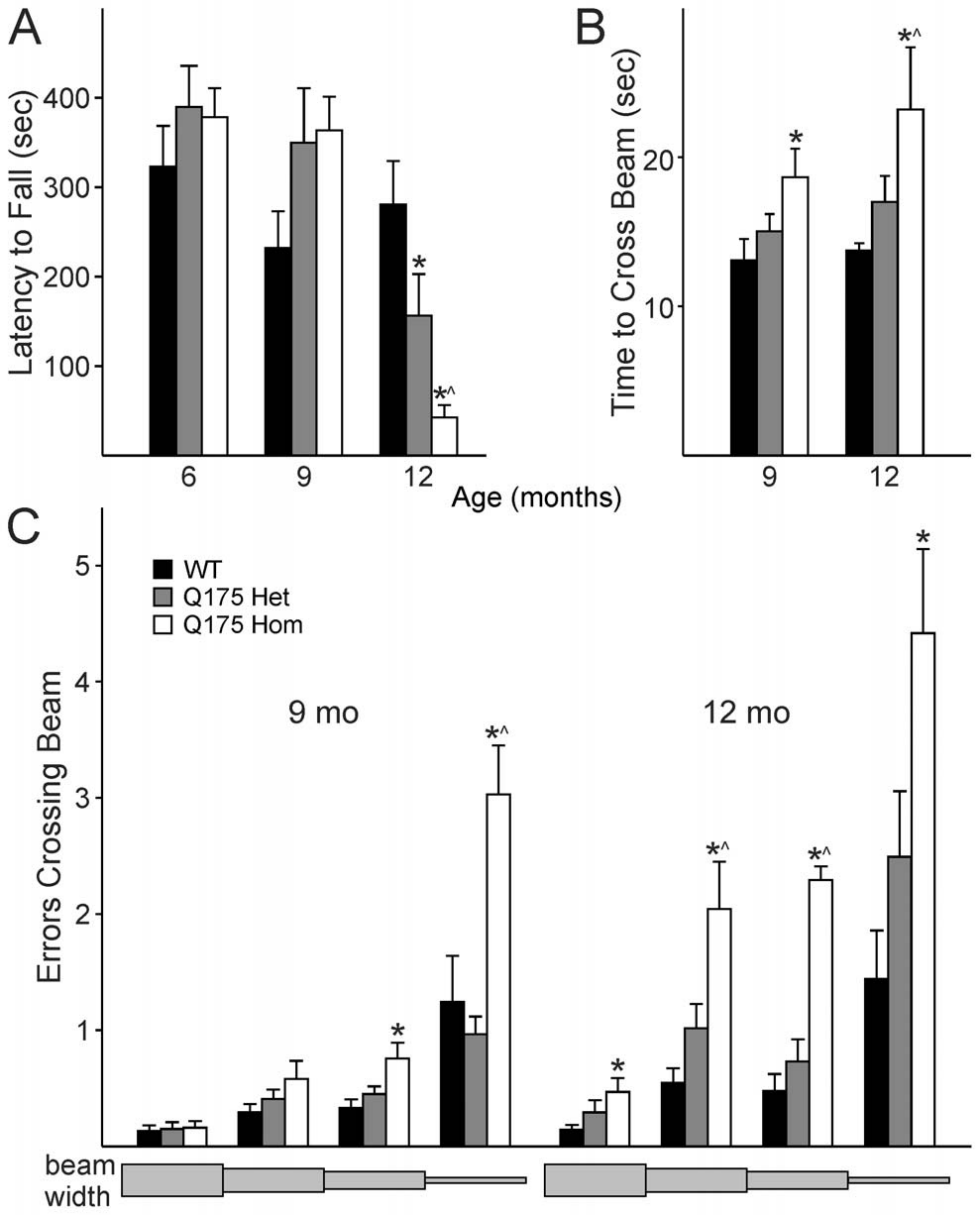
Motor ability of Q175 mutants declines with age. A. The accelerating rotarod test revealed that the age-related motor deficits in the Q175 model take up to 12 months to appear. B. 9 month old Q175 Hom mutants take longer to traverse the challenging beam test than age-matched WT and Q175 Het mice. C. The number of errors made while crossing each segment of the beam are shown, with the widest part of the beam on the left (33 mm) and narrowing towards the right (6 mm). The beam lengths are not to scale. The number of errors made while crossing the challenging beam are higher in Q175 Hom mutants. * indicates *P*<0.05 in *post hoc* pairwise comparisons with WT after an effect of genotype was detected by one way ANOVA, and ^∧^ indicates differences (*P*<0.05) between Q175 Het and Hom mice.

At 6 months of age, we assayed our cohort of WT, Q175 Het and Hom mice on the accelerating rotarod test, but found no significant effect of genotype on motor function as determined by latency to fall from the rotarod (*F*_2,22_ = 0.88, *P* = 0.430). Somewhat surprisingly, at 9 months, the Q175 Het and Hom mutants showed a trend towards better performance on the test than the WT controls (*F*_2,22_ = 2.935, *P* = 0.076), but this paradoxical finding was reversed by 12 months, where there was a gene dose-dependent decline in motor function (**Fig. 6A**; *F*_2,22_ = 11.557, *P*<0.001) with *post hoc* comparisons revealing that Het mice performed worse than WT (*t* = 2.419, *P* = 0.025) and Hom mice performed worse than both age-matched Het and WT mice (*vs* Het *t* = 2.225, *P* = 0.0378; *vs* WT *t* = 4.806, *P* = 0.017).

We employed the challenging beam test as a parallel test of the motor coordination at 9 months. We found clear motor deficits in the ability of Q175 Hom mice to cross the challenging beam at both 9 and 12 months. The Q175 Hom mice took longer to cross the beam at 9 and 12 months (Fig. 6B; 9 months *F*_2,22_ = 3.85, *P* = 0.039; 12 months *F*_2,18_ = 6.91, *P* = 0.007). The number of errors made by each Hom mouse were also considerably greater than those made by WT and Q175 Het mice at the same age (Fig. 6C; 9 months *F*_2,22_ = 13.569, *P*<0.001; 12 months *F*_2,18_ = 17.794, *P*<0.001). In the Q175 Hom group at 12 months, 4 of 8 mice failed to cross the beam successfully, and were not included further in the analysis.

The deterioration in challenging beam performance with age was tested using two way ANOVA, which did not reveal a significant effect of age on the time to cross the challenging beam (age *F*_1,41_ = 3.341, *P* = 0.076; genotype *F*_2,41_ = 10.761, *P*<0.001). On the other hand, the number of errors made while crossing the beam was both age- and genotype-dependent (age *F*_1,41_ = 31.575, *P*<0.001; genotype *F*_2,41_ = 32.741, *P*<0.001).

We can thus confirm that the Q175 Hom mice showed a decline in motor performance at 9 months as revealed by the challenging beam test, and that both Q175 Het and Hom mice showed deficits compared to age-matched WT controls on the accelerating rotarod test at 12 months.

### Sleep Analysis by Video Tracking Reveals Deficits in Temporal Patterning of Activity in Q175 Mutant Mice

**Figure 7:**
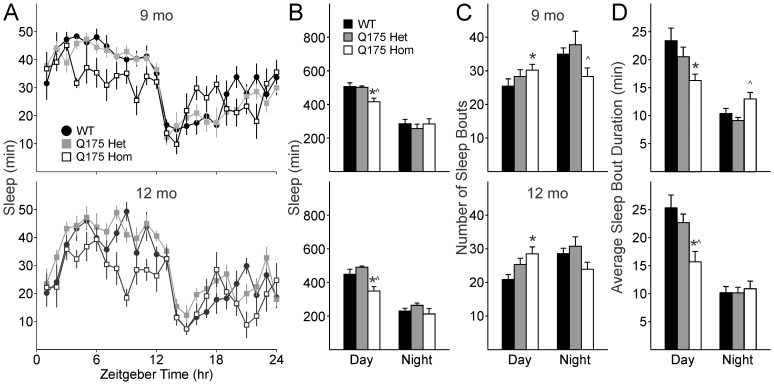
Daytime sleep is selectively affected in Q175 mutants. **A.** Average waveforms of hourly sleep in WT, Q175 Het and Hom mice at 9 months (top) and 12 months of age (bottom). **B.** Amount of time spent in sleep in day is selectively decreased compared to night-time sleep in Q175 Hom mice at 9 (top) and 12 months of age (bottom). **C.** 9 (top) and 12 month (bottom) old Q175 Hom mice have more fragmented daytime sleep, with increased number of sleep episodes. **D.** Sleep episodes in Q175 Hom mice are also shorter in duration. * indicates significant difference (*P*<0.05) between WT and Q175 Hom mice and ^∧^ indicates differences (*P*<0.05) between Q175 Het and Hom mice.

We used video recording to measure sleep as defined by time spent immobile, in combination with automated mouse tracking analysis software. Average waveforms of hourly immobility-defined sleep clearly show a change in the distribution of sleep in the Q175 Hom mice at both 9 (**Fig. 7A** **top**) and 12 months (**Fig. 7A** **bottom**). As nocturnal creatures, mice typically spend majority of the daylight hours inactive, and the reduction in nocturnality that we previously observed of Q175 Hom mutants using wheel running activity is replicated in these video measurements of freely behaving mice.

At 9 months, the Q175 Hom mutants spent less time in sleep during the day than WT and Q175 Het mice (**Fig. 7B** **top**; *F*_2,22_ = 8.85, *P* = 0.002), although nighttime sleep did not vary across the genotypes (*F*_2,22_ = 0.38, *P* = 0.686). Similarly, at 12 months, the Q175 Hom mutants spent less time in sleep during the day than age-matched WT and Q175 Het mice (**Fig. 7B** **bottom**; *F*_2,22_ = 11.42, *P*<0.001), with no significant effect of genotype during the night (*F*_2,22_ = 1.63, *P* = 0.222). An effect of age was confirmed by two way ANOVA comparing daytime sleep across both ages of all three genotypes (*F*_2,45_ = 8.12, *P* = 0.007) in addition to an effect of genotype (*F*_2,45_ = 1.13, *P*<0.001). *Post hoc* pairwise comparisons confirmed a significant difference between WT vs. Q175 Hom (*t*_15_ = 4.98, *P* = 0.025) and Q175 Het vs. Hom (*t*_14_ = 5.75, *P* = 0.017) in total daytime sleep duration.

To examine sleep quality in the Q175 model, fragmentation analysis was performed to determine the number of sleep bouts during both day and night, and the average duration of each bout of sleep. At 9 months, there was no significant difference in the number of daytime sleep bouts (**Fig. 7C** **top,** *F*_2,22_ = 1.78, *P* = 0.190). However, at 12 months of age, increased fragmentation becomes apparent (**Fig. 7C** **bottom,** *F*_2,22_ = 5.33, *P* = 0.014), where the Q175 Hom mice have significantly more daytime sleep bouts than WT mice (*t*_15_ = 3.25, *P* = 0.012). Duration of the average daytime sleep bout is selectively affected in Q175 mice at both 9 months (**Fig. 7D** **top**, *F*_2,22_ = 4.99, *P* = 0.020) and 12 months (**Fig. 7D** **bottom, Fig. S2**, *F*_2,22_ = 7.90, *P* = 0.003).

The impact of the Q175 mutation appears to have a selective effect on daytime sleep in the Hom mutants, with a specific reduction of the amount of time spent asleep in the day, as well as increased fragmentation of already reduced sleep.

### Unaltered Nissl+and PER2+Cells in the SCN of Q175 Mutant Mice

The pathophysiology of HD is neurodegeneration followed by neuronal cell death in vulnerable cell populations like the striatum. The CAG140 KI line exhibited loss of striatal neurons as the disease progressed [32], [33]. Striatal volume was found to be reduced in the Q175 Het and Hom mutant mice from 4 months [34]. We examined the SCN to ascertain the impact of the Q175 mutation on the neuronal cell counts in WT, Het and Hom mice.

**Figure 8:**
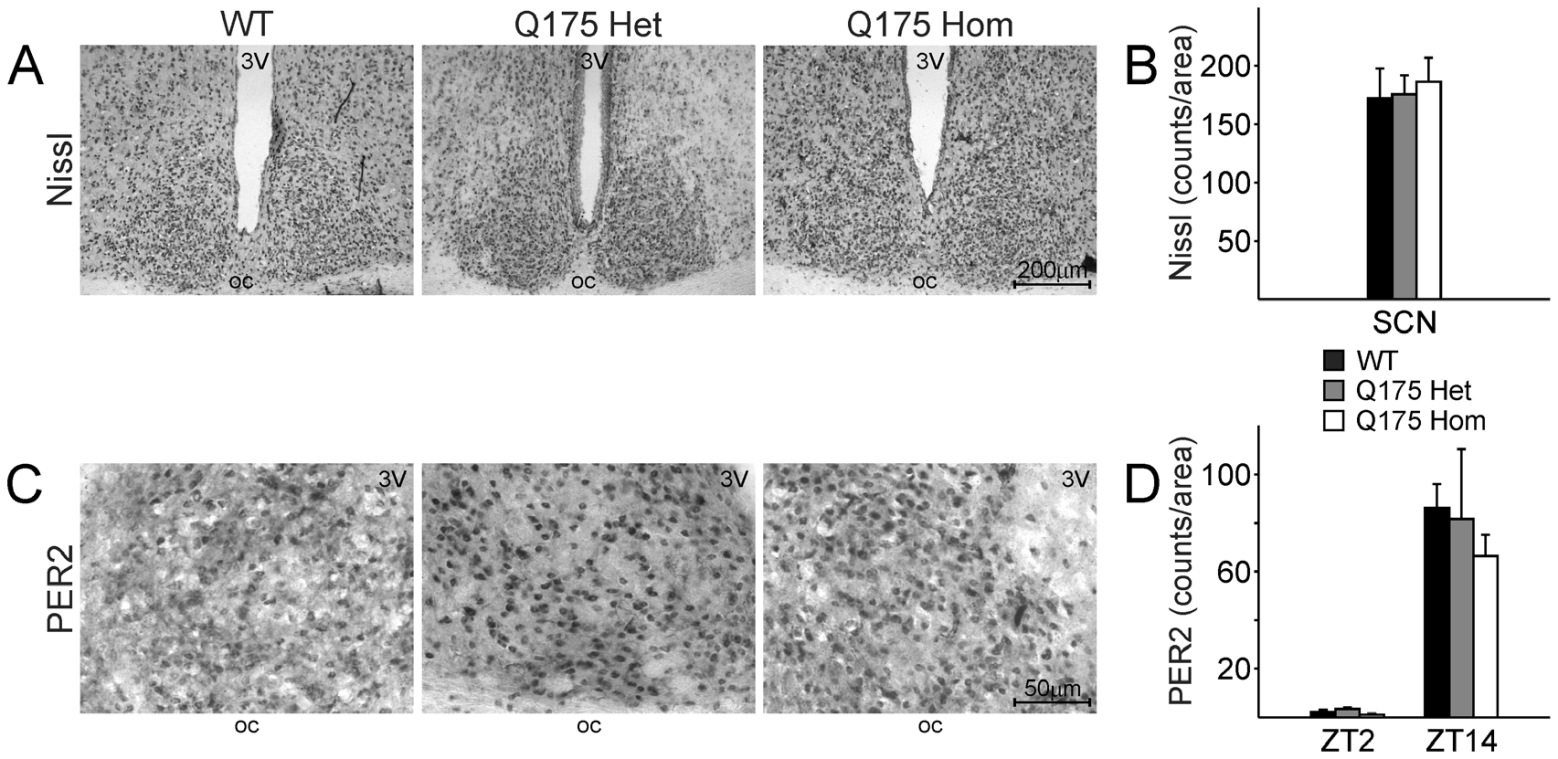
Neuron number and PER2 expression are unaffected in the SCN of Q175 mutants. **A.** Representative images of Nissl stains at 10X magnification of coronal sections of the SCN from WT (left), Q175 Het (middle) and Hom (right) mice. 3V: 3^rd^ ventricle. oc: optic chiasm. **B.** Quantification of Nissl+cells in the SCN from each genotype was determined by two independent observers blind to the experimental conditions. **C.** SCN expression of the circadian protein, PER2, is unaltered in Q175 mutants. Mice at 12m of age were sacrificed at ZT 2 and ZT 14, the respective trough and peak of SCN PER2 expression. Representative images of ZT 14 SCN, 40X magnification, are shown from each genotype. **D.** The day/night difference in PER2 expression is unaltered in Q175 mutants. Quantification of PER2+cells in the SCN was determined by 2 independent observers blind to the experimental conditions.

There were similar Nissl+cell counts in the SCN of all three genotypes (**Fig. 8A, 8B**; *F*_2,22_ = 0.105, *P* = 0.901). The size of the SCN was not different amongst the three groups (*F*_2,19_ = 0.053, *P* = 0.949). Examination of the lateral ventricles suggested that the Q175 Hom mice showed signs of brain volume loss (ANOVA *H*_19_ = 6.069, *P* = 0.048; *post hoc* comparison WT vs Hom *Q* = 2.409, *P*<0.05), in keeping with previous findings [**34**].

To determine the impact of the Q175 mutation on the molecular oscillator in the SCN, we examined the core circadian gene, PER2, at its peak (ZT 14, **Fig. 8C**) and trough (ZT 2) of expression using IHC. There was a time-of-day difference in each genotype as confirmed by two way ANOVA (**Fig. 8D**; *F*_1,29_ = 26.27, *P*<0.001). At ZT 2, the trough of PER2 expression, we found no significant effect of genotype (*F*_2,6_ = 1.774, *P* = 0.281). At ZT 14, the peak of PER2 expression, we also found no significant effect of genotype (*F*_2,11_ = 0.276, *P* = 0.765).

Our histological examination suggests that the Q175 mutation results in no obvious effect in the SCN. There is no apparent cell loss in the SCN at 13 months of age in the Q175 mutants. At the level of the molecular oscillator, the Q175 mutation does not affect the daily peak or trough of PER2 expression.

## Discussion

We found age- and genotype-dependent deterioration of the circadian parameters of activity, motor coordination and immobility-defined sleep in the Q175 mutant mice. It is intriguing that we observed greater deficits in the Q175 Hom than the Het mutants. HD in humans is a genetically dominant condition, but the gene dosage effect we and others [24], [34] observed in the Q175 line suggests that mice are more resistant to the same genetic load than humans. The more severe effect of a homozygous knocked-in HD mutation on motor function has also been observed in the CAG140 line [23] and similarly, transgenic YAC128 HD mice show a correlation between increased copy number and disease severity [35].

The decline in the circadian parameters of activity in the Q175 Hom mice was apparent at 9 months. In particular, the power of the rhythm under both LD and DD conditions dramatically dropped, similar to our previous findings of reduced power of locomotor rhythms in the BACHD model [20], albeit at a later age in the Q175 model. While these changes were concomitant with a decline in general locomotor activity, it is critical to note that precision of the daily onset of activity also deteriorated at the same time and suggests the circadian disruption is not simply due to motor dysfunction. The decline in precision and power of rhythms suggests an intrinsically weaker circadian oscillator, which also manifested in a change in the temporal pattern of the activity. The percentage of daily activity conducted in the night decreased with age to a greater degree in the Q175 Hom than the Het and WT mice. We observed a similar loss of temporal patterning of heart rate and body temperature in the BACHD model, which suggests that the circadian deficits also affect non-motor systems in HD.

It is also important to note that the Q175 Het and Hom mutants had normal performance on the rotarod test at the same age as the onset of the circadian deficits, suggesting that not all of the wheel running deficits we observed could be ascribed to an inability to turn the wheel at 9 months of age. However, by 12 months of age, Q175 Het and Hom mutants lose the motor coordination needed to perform the rotarod test, as has been documented in a separate cohort of Q175 mutants [24]. Further evidence of motor deficits include an increase in errors made by the Q175 Hom mutants while crossing the challenging beam, which could in part be explained by decreased grip strength from an early age [23].

In a video-based assay that is independent of the animal’s ability to turn a wheel, we assessed the sleep behavior of the mice, and found that daytime sleep was selectively affected by the Q175 mutation. While video analysis of sleep does not allow us to draw conclusions regarding the depth of sleep nor the relative progression from one state of sleep to another (e.g. NREM vs. REM), such measurements have been shown to accurately measure sleep and correlate highly with simultaneously recorded EEG-defined sleep [31], [36]. Using immobility-defined sleep analysis, we found that the Q175 Hom mice spent considerably less time asleep during the day than their WT and Het age-matched cohort. This is reflected by the reduced nocturnality that we observed in the wheel running assay, confirming that the temporal patterning of activity and inactivity is aberrant in these mutants. We also found increased fragmentation in daytime sleep in the Q175 Hom mice, which had an increased number of sleep bouts coupled with shorter sleep bouts than Het and WT mice at both 9 and 12 months of age. This fragmentation is in common with the sleep disturbances observed in HD patients, who suffer from reduced nighttime sleep efficiency (e.g. [12]).

The aberrant circadian locomotor activity and reduced daytime sleep that we observe suggest that an upstream regulator of behavior is affected, and the SCN as the master pacemaker is the most likely suspect. The SCN is dependent on intrinsic molecular oscillators within each cell that are governed by a core set of genes in a transcription-translation-post-translational feedback loop that takes ∼24 hr to complete each cycle (reviewed in [37]). Examination of the rhythms of PER2 expression in the SCN suggests that the deficit does not lie in the molecular oscillator within the SCN, although other components of the oscillator may be affected. However, an intact molecular oscillator in the SCN may not be indicative of a normally functioning circadian system. We have now consistently seen a dissociation between normal PER2 rhythms in the SCN and reduced SCN output in three different models of neurodegeneration: the BACHD mouse model, a mouse model of Parkinson’s Disease, and in middle-aged mice [20], [38], [39]. In the R6/2 model, the HD mutation had a stronger impact on the non-SCN circadian oscillators, suggesting the output mechanisms by which the SCN communicates are affected [10], [40], [41].

Likely candidates for the decline in circadian output from the SCN include deficits in neuron-to-neuron communication. For example, neuropeptides critical for communication within the circadian circuitry are selectively lost in the SCN of HD patients [42]. Prior work has also demonstrated a loss of one of those peptides, vasoactive intestinal peptide (VIP), in the SCN neurons of the R6/2 model [43]. VIP has been shown to be critical for robust rhythms within the SCN and for its output [25], [44]–[46]. A reduction in VIP would result in a weakened SCN output, and is consistent with the circadian phenotype that we observed in the BACHD and now in the Q175 model.

Steps towards stabilizing rhythms in the R6/2 mouse model, e.g. scheduling meal times and imposing sleep during the day, suggest that there is merit in pursuing treatment strategies for the non-motor symptoms of HD, even to the extent of delaying the progression of motor dysfunction and improving cognitive function [40], [41], [47]. It is thus critical to determine the relative contributions of disrupted rhythms versus neurodegeneration in the motor control system on cognitive, psychiatric and metabolic dysfunction, which would suggest better targets for therapy. The Q175 model is a particularly salient model to study due to the precise insertion of HD mutation into the mouse *Htt* locus and the relatively similar progression of symptoms to HD patients [24], [34]. The sleep and circadian rhythm disruptions observed in the Q175 line make it a promising model in which to determine how the genetic cause of HD leads to disrupted rhythms.

## Supporting Information

Figure S1 **Comparison of immobility-defined sleep at different thresholds of immobility detection.** **A.** Amount of sleep during the day and night in 12 month old WT, Q175 Het and Q175 Hom mice under the three different immobility detection thresholds. At all three detection thresholds, the Q175 Hom mice exhibit reduced daytime sleep compared to WT mice (* *P*<0.05 vs. WT, ^∧^ *P*<0.05 vs. Het). **B.** Average sleep waveforms do not change in shape or direction of difference under the three different immobility thresholds.
(TIF)

Figure S2 **Distribution of daytime sleep bout duration is altered inQ175 Hom mice.** **A.** Sleep bouts of 30 min intervals were normalized to the total number of bouts in the day. Short bouts of under 30 min duration were increased, and longer bouts were decreased in Q175 Hom mice (* *P*<0.05 vs. WT, ^∧^ *P*<0.05 vs. Het). **B.** Higher resolution display of the distribution of sleep bout durations shorter than 30 min, normalized to the total number of bouts during the day.
(TIF)

Table S1 **Comparison of immobility-defined sleep at three thresholds of immobility detection: 90, 95 and 97%.** The differences in sleep measured at the 90% and 97% immobility detection settings compared to sleep at the 95% setting are reported as a percentage (Δ). The effect of genotype was determined using one-way ANOVA, and the *F* and *P* statistics are reported for genotype comparisons. * indicates that *post hoc* Bonferroni’s *t*-tests detected significant differences compared to WT and ^#^ indicates differences between Het and Hom.
(DOCX)

Text S1 **Sleep and circadian deficits in the Q175 HD model - Supplemental Materials.**
(DOCX)
