# Supplementary figures and images for: The Q175 Mouse Model of Huntington’s Disease Shows Gene Dosage- and Age-Related Decline in Circadian Rhythms of Activity and Sleep
Source: PLoS One. 2013 Jul 30;8(7):e69993. doi: 10.1371/journal.pone.0069993 (PMC3728350; doi:10.1371/journal.pone.0069993)

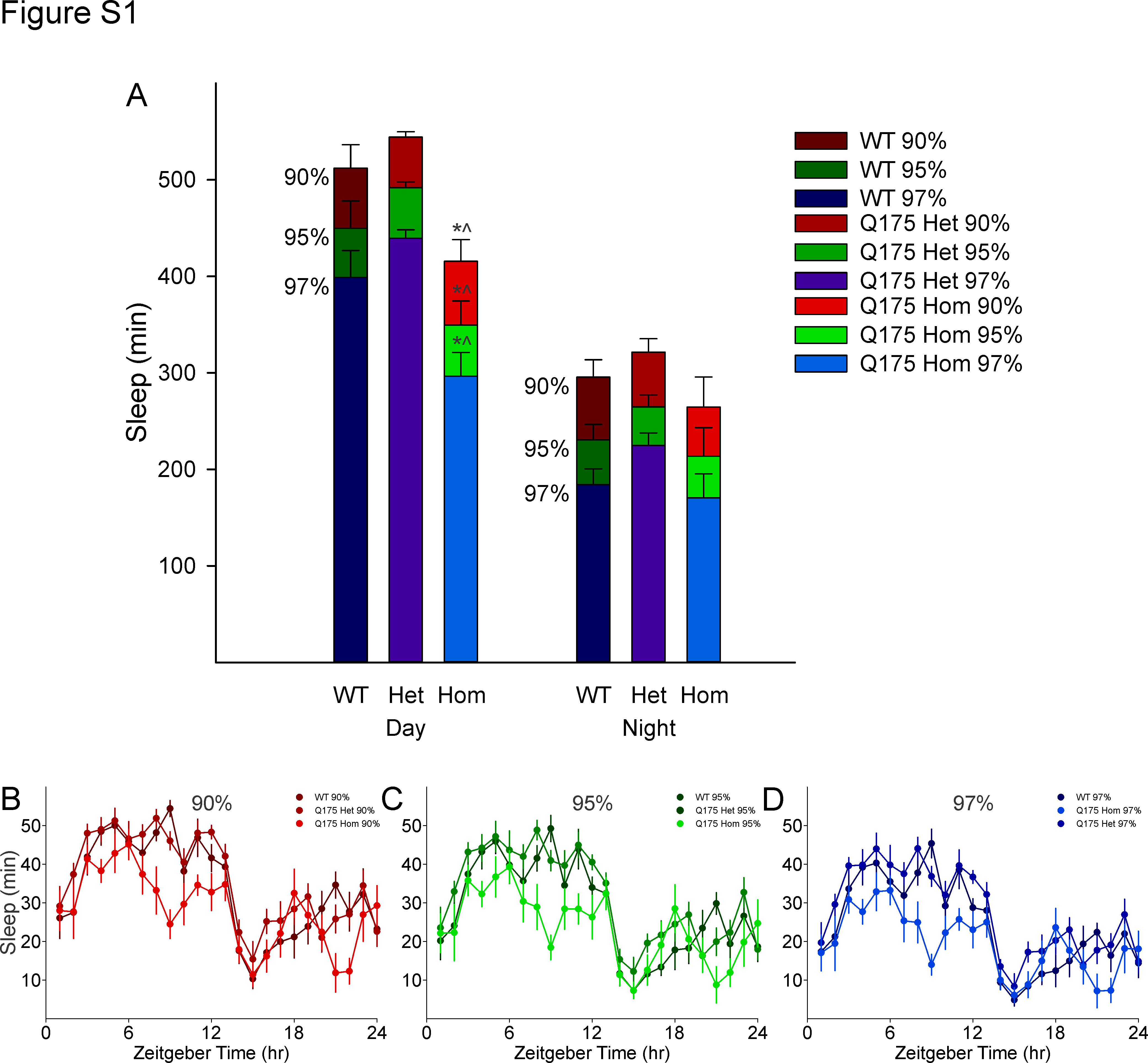

Supplement: Figure S1 — Comparison of immobility-defined sleep at different thresholds of immobility detection. A. Amount of sleep during the day and night in 12 month old WT, Q175 Het and Q175 Hom mice under the three different immobility detection thresholds. At all three detection thresholds, the Q175 Hom mice exhibit reduced daytime sleep compared to WT mice (* P<0.05 vs. WT, ∧ P<0.05 vs. Het). B. Average sleep waveforms do not change in shape or direction of difference under the three different immobility thresholds. (TIF) [file pone.0069993.s001.tif]

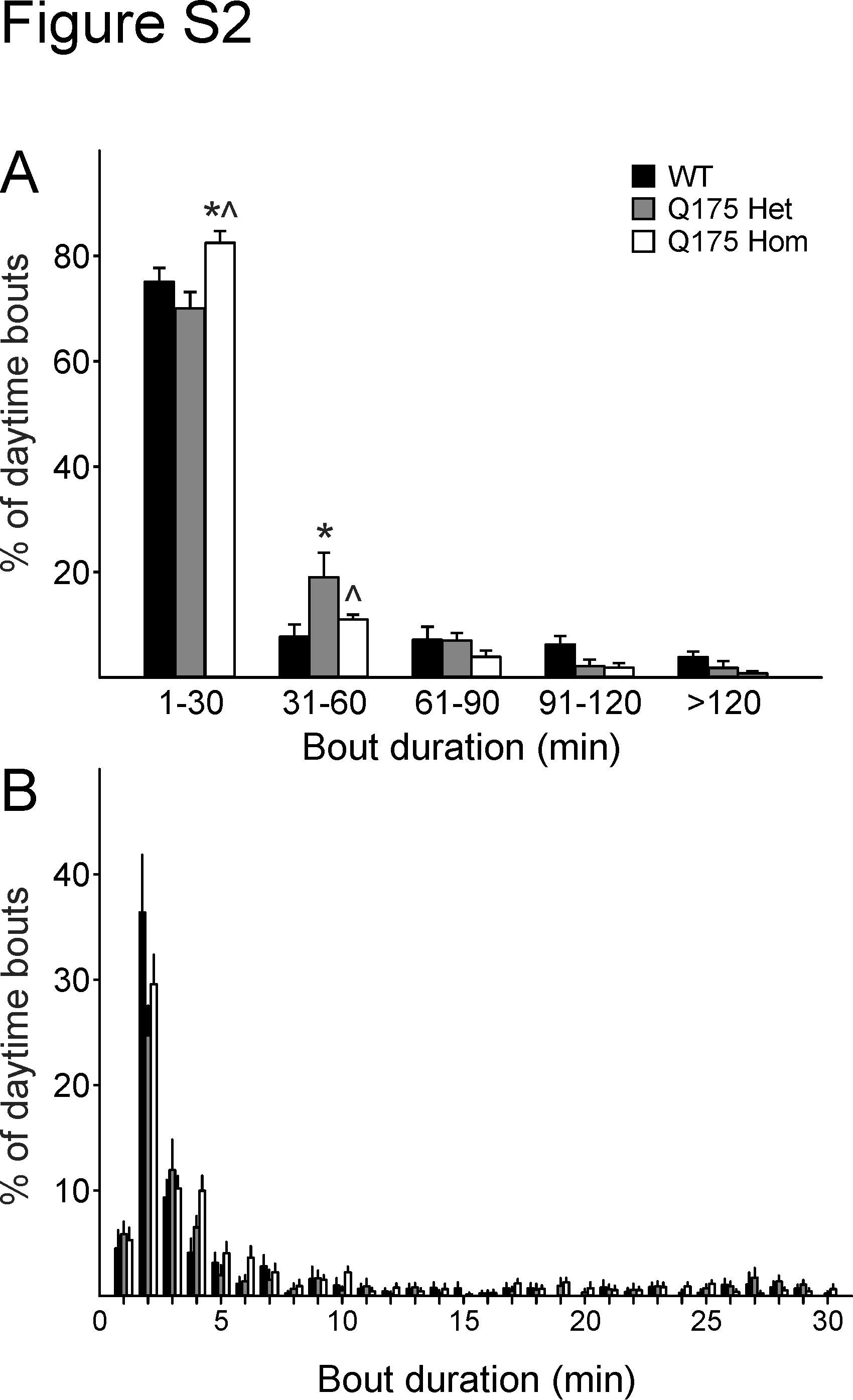

Supplement: Figure S2 — Distribution of daytime sleep bout duration is altered inQ175 Hom mice. A. Sleep bouts of 30 min intervals were normalized to the total number of bouts in the day. Short bouts of under 30 min duration were increased, and longer bouts were decreased in Q175 Hom mice (* P<0.05 vs. WT, ∧ P<0.05 vs. Het). B. Higher resolution display of the distribution of sleep bout durations shorter than 30 min, normalized to the total number of bouts during the day. (TIF) [file pone.0069993.s002.tif]
