# Supplementary material for: The Q175 Mouse Model of Huntington’s Disease Shows Gene Dosage- and Age-Related Decline in Circadian Rhythms of Activity and Sleep
Source: PLoS One. 2013 Jul 30;8(7):e69993. doi: 10.1371/journal.pone.0069993 (PMC3728350; doi:10.1371/journal.pone.0069993)
Supplement: Table S1 — Comparison of immobility-defined sleep at three thresholds of immobility detection: 90, 95 and 97%. The differences in sleep measured at the 90% and 97% immobility detection settings compared to sleep at the 95% setting are reported as a percentage (Δ). The effect of genotype was determined using one-way ANOVA, and the F and P statistics are reported for genotype comparisons. * indicates that post hoc Bonferroni’s t-tests detected significant differences compared to WT and # indicates differences between Het and Hom. (DOCX) [file pone.0069993.s003.docx]

Table S1

|  | **WT** | | **Q175 Het** | | **Q175 Hom** | | **One way ANOVA** | |
| --- | --- | --- | --- | --- | --- | --- | --- | --- |
|  | Day (min) | Δ | Day (min) | Δ | Day (min) | Δ | *F* | *P* |
| 90% threshold | 512 ± 24 | +14% | 544 ± 5 | +10% | 416 ± 23*^#^ | +19% | 12.57 | <0.001 |
| 95% threshold | 450 ± 29 |  | 492 ± 6 |  | 350 ± 25*^#^ |  | 11.42 | <0.001 |
| 97% threshold | 399 ± 28 | -11% | 439 ± 9 | -11% | 297 ± 25*^#^ | -20% | 11.78 | <0.001 |
|  |  |  |  |  |  |  |  |  |
|  | Night (min) | Δ | Night (min) | Δ | Night (min) | Δ |  |  |
| 90% threshold | 296 ± 18 | +28% | 322 ± 14 | 21% | 265 ± 31 | +24% | 1.73 | 0.2 |
| 95% threshold | 230 ± 16 |  | 265 ± 13 |  | 213 ± 30 |  | 1.63 | 0.22 |
| 97% threshold | 184 ± 16 | -20% | 225 ± 13 | -15% | 170 ± 25 | -20% | 2.38 | 0.12 |
