# Supplementary material for: The Q175 Mouse Model of Huntington’s Disease Shows Gene Dosage- and Age-Related Decline in Circadian Rhythms of Activity and Sleep
Source: PLoS One. 2013 Jul 30;8(7):e69993. doi: 10.1371/journal.pone.0069993 (PMC3728350; doi:10.1371/journal.pone.0069993)
Supplement: Text S1 — Sleep and circadian deficits in the Q175 HD model - Supplemental Materials. (DOCX) [file pone.0069993.s004.docx]

Sleep and circadian deficits in the Q175 HD model - Supplemental Materials

***Sensitivity of software detection of immobility***

To address the possibility that the motor dysfunction in the Q175 mutants affects the sensitivity of the software analysis of immobility-defined sleep, we performed the analysis at two additional thresholds of immobility detection previously shown by Fisher and colleagues [1] to over- and under-estimate sleep when compared to concurrent EEG recordings. The 90% threshold was shown to over-estimate sleep by 20% and the 97% threshold was shown to under-estimate sleep by 20%. We compared the immobility-defined sleep from these two thresholds to the recommended immobility detection setting of 95%. We found that the amount of immobility-defined sleep increased on application of the 90% threshold and decreased on application of the 97% threshold in all three genotypes (**Table S1**). Crucially, within each threshold, the differences between the genotypes were maintained. The finding of reduced amount of sleep in the day and night in Q175 Hom mice was consistent through the different settings (**Fig. S1A**). The daily profile of sleep was also maintained at the different immobility settings (**Fig. S1B**).

***Sleep bout durations measured by immobility-defined methods are comparable to sleep bouts/epochs determined by EEG recording in the current literature***

To address concerns that the immobility-defined sleep bouts we measured are reflective of sleep bouts measured by more traditional EEG methods, we examined sleep at 1 min resolution intervals. We were limited in our approach compared to the second-by-second resolution obtained using EEG as we wished to err on the side of caution and maintain the minimum of 40 seconds of immobility previously determined by Pack and colleagues [2] to have the smallest prediction error of sleep. Regardless, we found that the majority of sleep bouts defined by our analysis were of short duration (<30 min, **Fig. S2A**). Furthermore, the distribution of sleep bouts was similar to the distribution reported in the study by Pack and colleagues [2] using both video-based and EEG methodology, and other recent publications examining sleep bout durations using EEG alone [e.g. 3]. In both studies, the majority of sleep was recorded between 1 and 2 min in duration, and our analysis of sleep bout duration we recorded is similar in distribution (**Fig. S2B**).

**References**

1. Fisher SP, Godinho SIH, Pothecary CA, Hankins MW, Foster RG, et al. (2012) Rapid assessment of sleep-wake behavior in mice. J Biol Rhythms 27: 48–58. doi:10.1177/0748730411431550.

2. Pack AI, Galante RJ, Maislin G, Cater J, Metaxas D, et al. (2007) Novel method for high-throughput phenotyping of sleep in mice. Physiol Genomics 28: 232–238. doi:10.1152/physiolgenomics.00139.2006.

3. Mochizuki T, Crocker A, McCormack S, Yanagisawa M, Sakurai T, et al. (2004) Behavioral state instability in orexin knock-out mice. J Neurosci 24: 6291–6300. doi:10.1523/JNEUROSCI.0586-04.2004.
